# Modified APPEND Score for the Diagnosis of Acute Appendicitis in a New Zealand Pasifika Population

**DOI:** 10.1002/wjs.12510

**Published:** 2025-02-19

**Authors:** Renato Pitesa, Andrew G. Hill, Andrew D. MacCormick

**Affiliations:** ^1^ Department of Surgery The University of Auckland Auckland New Zealand; ^2^ Department of Surgery Middlemore Hospital Auckland New Zealand

**Keywords:** appendicitis, low‐ and middle‐income countries, pacific

## Abstract

**Background:**

Diagnosing acute appendicitis often requires biochemical and imaging support which may not be feasible in low‐ and middle‐income countries (LMICs). The APPEND score, developed in New Zealand, includes C‐reactive protein (CRP) which in resource‐limited settings, may be hindered by slow processing times. This study aims to evaluate a modified APPEND score (mAPPEND), excluding CRP for diagnosing appendicitis in a New Zealand Pasifika cohort.

**Methods:**

This secondary analysis utilized data from two cohorts (2011 and 2017) from Middlemore Hospital, Auckland. Patients aged ≥ 15 years with right iliac fossa pain for < 7 days were included, excluding those with prior appendicectomy or generalized peritonitis. Sensitivity, specificity, positive predictive value (PPV), and negative predictive value (NPV) were calculated, and diagnostic performance was assessed using receiver operating characteristic curve analysis, comparing the area under the curve (AUC) for both scores.

**Results:**

Among 143 Pasifika patients, the AUC for the APPEND and mAPPEND scores were comparable (0.84 vs. 0.85 respectively, *p* = 0.41). The mAPPEND score demonstrated high diagnostic accuracy with scores between 1 and 2 showing high sensitivity (100% and 97%) and NPV (90% and 92%), scores 4–5 showing high specificity (94% and 100%, respectively) and PPV (90% and 100%, respectively), and a score of 3 being the most efficient with a sensitivity of 82% and specificity of 71%.

**Conclusion:**

The mAPPEND score maintains high diagnostic accuracy for appendicitis in a New Zealand Pasifika population. This modified score is a simple and viable tool in settings where CRP testing is unfeasible, supporting its use in Pacific Island countries.

## Graphical abstract

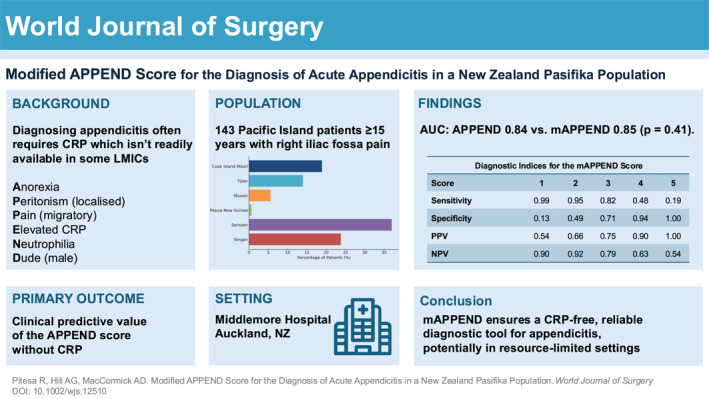

## 1 Introduction

The diagnosis of acute appendicitis is largely clinical, but establishing a diagnosis based on clinical presentation and examination alone is often difficult [1]. Reliance on biochemical and imaging modalities has increased, with some guidelines recommending standard imaging in all potential appendicitis patients [2]. In low‐ and middle‐income countries (LMIC) or resource‐constrained settings, this is not practical, and a different approach is needed because of the absence of prompt laboratory or radiology services. Disparities in access to such modalities can delay the timely diagnosis of acute presentations, impeding the delivery of quick and effective management.

Clinical prediction rules (CPRs) have emerged as essential tools used to help guide medical decision‐making, particularly in LMIC, and there are a plethora of scoring tools available to help risk stratify appendicitis patients [3]. For example, the APPEND score is a diagnostic tool developed at Middlemore Hospital in South Auckland, New Zealand, to assist in the diagnosis and management of patients presenting with right iliac fossa pain [4]. Although demonstrated to be valuable in Middlemore Hospital, the APPEND score requires access to CRP, a biochemical marker of inflammation, which in many LMICs within the South Pacific is limited by slow laboratory processing times, variable results, and lack of specificity [5]. For instance, Rivara et al. found that like other populations, baseline CRP levels in Samoans differed by sex, adiposity, and cardiometabolic risk factors [6].

There are several CPR's readily available to assist in the diagnosis of appendicitis including the Alvarado score, the Appendicitis Inflammatory Response Score (AIRS), and the Lintula score [3]. However, there is evidence to suggest that in certain geographical or ethnic populations, the diagnostic performance of certain scores do not perform as well as hoped. For example, the Alvarado score has found to be a relatively poor predictor of appendicitis in Asian and Middle Eastern populations in comparison with the RIPASA score [7]. In addition to this, evidence on intrabdominal infections in Pacific Island populations is scarce in comparison to other global regions. For this matter, this study aims to reevaluate the APPEND score, omitting CRP testing, to determine its efficacy in a New Zealand‐based Pasifika cohort.

## 2 Methods

### 2.1 Study Design/Participants

This study was a secondary analysis of two previously published cohorts that examined the diagnostic accuracy of the APPEND score for acute appendicitis. The two cohorts were comprised of a retrospective cohort from 2011 and a prospective cohort from 2017, both from Middlemore Hospital, Auckland, New Zealand, a tertiary‐level hospital [4, 8]. Ethics approval was obtained for both original studies and this secondary analysis (Auckland Health Research Ethics Committee #AH26893). The study included patients aged ≥15 years who presented with right iliac fossa pain for < 7 days, excluding those with a history of appendicectomy or generalized peritonitis. Demographic information, clinical symptoms, and laboratory test results were collected from patients' medical records.

### 2.2 Variables/Outcomes

The APPEND score was calculated based on six original variables: anorexia, migratory pain, peritonism, elevated CRP, neutrophilia, and male sex. For the modified score, CRP was omitted.

The primary outcome was to determine the clinical predictive value (sensitivity, specificity, positive predictive value, and negative predictive value) for both the APPEND and modified APPEND scores in a Pacific Island population. The secondary outcome was the presence of appendicitis, which was histologically defined after appendicectomy, and rates of complicated appendicitis as defined by the American Association for the Surgery of Trauma (AAST) grading system [9].

### 2.3 Statistical Analysis

Receiver operating characteristic (ROC) curve analysis was used to evaluate the diagnostic performance of the APPEND and modified APPEND (mAPPEND) scores. The area under the curve (AUC) was calculated for each score and statistical comparisons were performed using DeLong's test for paired ROC curves. Optimal threshold scores were determined by identifying the threshold closest to the ideal point (sensitivity = 1, specificity = 1) on the ROC curve. Diagnostic indices including sensitivity, specificity, positive predictive value (PPV), negative predictive value (NPV), and efficiency were calculated at each threshold for both scores utilizing confusion matrices. Statistical analyses were conducted using R studio software (version 4.3.2; R Studio, Boston, MA), with the “pROC” and “caret” packages.

## 3 Results

### 3.1 Patient Characteristics

From a collated database of 643 patients, 143 of those identified as Pacific Islander and were included in the final analysis. The study consisted of participants aged 15–71 years (mean 31.8 [14.5]; median 27 [20–43.5]) with a female preponderance (57.3%). There were no statistically significant differences at the baseline between appendicitis and non‐appendicitis cases (Table 1). Of those diagnosed with appendicitis, 23.3% of those were considered complicated at the time of operation with no statistically significant differences seen between ethnicities (Table 2).

**TABLE 1:** Sample population demographics.

	Appendicitis		
Characteristic	No	Yes	*p*‐value^b^
	*N* = 70^a^	*N* = 73^a^	
Age	30 (14)	33 (15)	0.2
Sex			0.020
Female	47 (67%)	35 (48%)	
Male	23 (33%)	38 (52%)	
Ethnicity			0.4
Cook island Māori	12 (17%)	15 (21%)	
Fijian	11 (16%)	9 (12%)	
Niuean	6 (8.6%)	2 (2.7%)	
Papua New Guinean	1 (1.4%)	0 (0%)	
Samoan	22 (31%)	31 (42%)	
Tongan	18 (26%)	16 (22%)	

^a^ Mean (SD); *n* (%).

^b^ Welch Two Sample *t*‐test; Pearson’s Chi‐squared test; Fisher’s exact test.

**TABLE 2:** Rates of complicated appendicitis and AAST grade of those with histologically confirmed appendicitis.

		Confirmed appendicitis					
Characteristic	Cook island Māori	Fijian	Niuean	Samoan	Tongan	Overall	*p*‐value^a^
	*n* = 15	*n* = 9	*n* = 2	*n* = 31	*n* = 16	*n* = 73	
Complicated							> 0.9
No	12 (80%)	7 (78%)	2 (100%)	23 (74%)	12 (75%)	56 (76.7%)	
Yes	3 (20%)	2 (22%)	0 (0%)	8 (26%)	4 (25%)	17 (23.3%)	
AAST grade							> 0.9
1	12 (80%)	7 (78%)	2 (100%)	23 (74%)	12 (75%)	56 (76.7%)	
2	1 (6.7%)	1 (11%)	0 (0%)	2 (6.5%)	0 (0%)	4 (5.5%)	
3	1 (6.7%)	0 (0%)	0 (0%)	2 (6.5%)	1 (6.3%)	4 (5.5%)	
4	1 (6.7%)	1 (11%)	0 (0%)	4 (13%)	3 (19%)	9 (12.3%)	
5	0 (0%)	0 (0%)	0 (0%)	0 (0%)	0 (0%)	0 (0%)	

^a^ Fisher’s exact test.

### 3.2 Diagnostic Performance of the Scores

**FIGURE 1:**
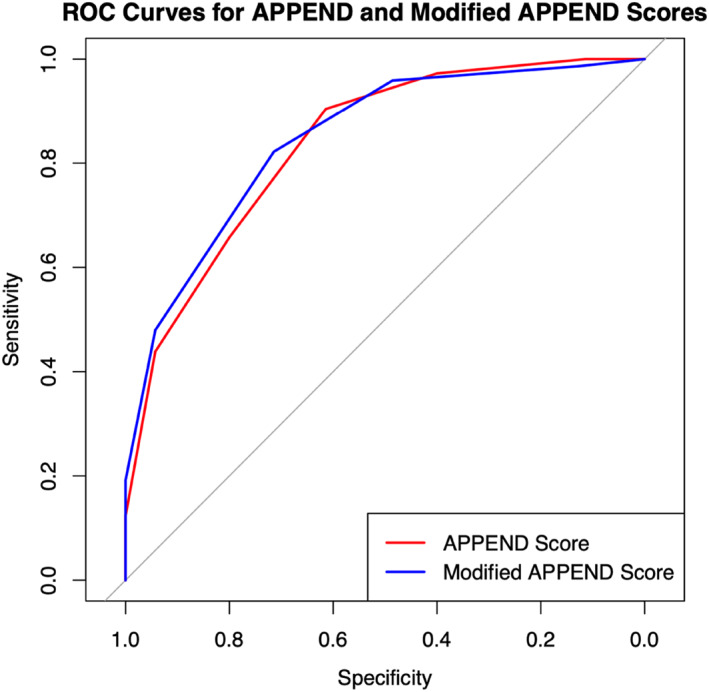
ROC curves for APPEND and mAPPEND scores.

The AUC for the APPEND and mAPPEND scores were comparable, with no statistically significant difference (0.84 [95% CI: 0.77–0.90] vs. 0.85 [95% CI 0.79–0.91] *p* = 0.41) (Figure 1).

The diagnostic indices for APPEND and mAPPEND scores in this cohort are shown in Tables 3 and 4, respectively. Modified APPEND scores between 1 and 2 had high sensitivity (100% and 97%) and NPV (90% and 92%). Scores between 4 and 5 demonstrated high specificity (94% and 100%, respectively) and PPV (90% and 100%, respectively), with a score of 5 having a no negative appendicectomy cases (Table 5). A score of 3 was found to have a balanced diagnostic performance, with a sensitivity and specificity of 82% and 71%, and NPV and PPV of 79% and 75%, respectively.

**TABLE 3:** Diagnostic indices for the APPEND score.

Score	Diagnostic indices for the APPEND score					
	1	2	3	4	5	6
Sensitivity	1.00	0.97	0.90	0.66	0.44	0.12
Specificity	0.11	0.4	0.61	0.80	0.94	1.00
PPV	0.54	0.63	0.71	0.77	0.88	1.00
NPV	1.00	0.93	0.86	0.69	0.62	0.52
Efficiency	0.57	0.69	0.76	0.73	0.69	0.55

**TABLE 4:** Diagnostic indices for the modified APPEND score.

Score	Diagnostic indices for the mAPPEND score				
	1	2	3	4	5
Sensitivity	0.99	0.95	0.82	0.48	0.19
Specificity	0.13	0.49	0.71	0.94	1.00
PPV	0.54	0.66	0.75	0.90	1.00
NPV	0.90	0.92	0.79	0.63	0.54
Efficiency	0.57	0.72	0.77	0.70	0.59

**TABLE 5:** Number of participants with and without appendicitis per score.

	Appendicitis			
mAPPEND score	No, *n* = 70^a^	Yes, *n* = 73^a^	Overall, *n* = 143^a^	*p*‐value^b^
mAPPEND score				< 0.001
0	9 (13%)	1 (1.4%)	10 (7.0%)	
1	25 (36%)	2 (2.7%)	27 (19%)	
2	16 (23%)	10 (14%)	26 (18%)	
3	16 (23%)	25 (34%)	41 (29%)	
4	4 (5.7%)	21 (29%)	25 (17%)	
5	0 (0%)	14 (13%)	14 (9.8%)	

^a^ *n* (%).

^b^ Fisher's exact test.

## 4 Discussion

This was a retrospective secondary analysis of two separate single‐center cohorts to ascertain the diagnostic utility of the mAPPEND score in a Pasifika context. Our findings showed that in a Pasifika population, the APPEND score had excellent diagnostic accuracy (AUC: 0.83 [95% CI: 0.73–0.94]), and that while CRP is valuable, it was not needed to achieve robust diagnostic performance. Scores ≥ 4 reliably indicate the presence of appendicitis and scores ≤ 2 effectively rule it out, leaving a score of 3 which may prompt additional diagnostic evaluation contingent on the availability of resources.

The performance of the mAPPEND score in this study emphasizes the importance of validating CPRs across diverse ethnic and geographical populations. As mentioned previously, diagnostic tools developed in high income western populations may not easily generalize to LMIC leading to possible underperformance in such contexts like the Alvarado score in Asia and the Middle East [7]. Similarly, Pasifika populations may present with unique pathophysiological patterns influenced by genetic, dietary, environmental, and financial factors. For example, Pifeleti et al. found that almost 70% of patients in Samoa presenting to hospital with appendicitis were perforated at the time of operation, which is contrasted to our findings of only 23.3% and those of de Burlet et al. reporting a complicated appendicitis rate of 22.7% in Aotearoa New Zealand [10, 11]. The findings in Samoa are consistent in other LMICs with reported complicated appendicitis rates of up to 60% in Ethiopia, 80% in South Africa, and 80% in Bangladesh [12, 13, 14]. Moreover, within Aotearoa New Zealand, incidence of appendicitis did not vary greatly between ethnicities, but Māori and Pacifika adults trended toward higher rates of complicated appendicitis irrespective of delayed presentation [15]. It is well known that complicated appendicitis (American Association for the Surgery of Trauma [AAST] grade ≥ 2) is associated with increased morbidity and mortality and implementing minimal‐cost scoring systems like the mAPPEND score could expedite definitive management and improve patient outcomes [9, 16].

Furthermore, the findings of this study have important implications in reducing negative appendicectomy rates. Negative appendicectomy is associated with unnecessary hospital costs in addition to potentially increased morbidity and mortality [17]. A study from Papua New Guinea found that the cost of a negative appendicectomy admission was comparable to the cost of an uncomplicated appendicitis (US$11,263.25 vs. US$11,460.12) [18]. In the prospective validation of the APPEND score, it was found that negative appendicectomy rates were halved from 19.8% to 9.2% [4]. The high NPV and sensitivity at lower thresholds and reciprocal increase of PPV and specificity at high thresholds show promise at minimizing unnecessary operations and missed diagnoses. For those with an intermediate score of 3, a pragmatic approach would involve admitting for the observation of clinical parameters, repeat biochemical tests, and further radiological investigation with point‐of‐care ultrasound in the first instance. Patients with inconclusive ultrasound findings may benefit from a low‐dose contrast‐enhanced CT scan prior to surgery if resources allow [1].

This study has several limitations. First, the reduced sample size limits the statistical power of the results, reducing the likelihood of detecting subtleties within diagnostic performance. Second, approximately half of the participants were drawn from a cohort that was retrospectively collected, which introduced a set of information and sampling biases. The retrospective nature of this analysis made it impossible to ensure complete and consistent data documentation. Finally, as the scope of this analysis was specific to the Pasifika population, the generalizability to other groups or regions is limited. In saying that, the original APPEND score was validated at a center which serves an ethnically diverse population [19].

## 5 Conclusion

In conclusion, the omission of CRP from the APPEND score does not compromise the overall effectiveness of the tests in the Pasifika context and, in fact, provides better clinical predictive values when compared to the APPEND score. The comparable diagnostic accuracy to the original score supports its adoption as a viable diagnostic tool in the Pasifika setting. This needs to be confirmed in patients based in their respective Pacific Island countries. Further large, multicenter prospective validation studies are required to establish its widespread applicability and support its successful implementation in clinical practice.

## Author Contributions

**Renato Pitesa:** data curation, formal analysis, methodology, resources, visualization, writing–original draft. **Andrew G. Hill:** conceptualization, project administration, supervision, writing–review & editing. **Andrew D. MacCormick:** conceptualization, methodology, project administration, supervision, writing–review & editing.

## Conflicts of Interest

The authors declare no conflicts of interest.
